# A Patient Perspective on Seizure Detection and Forecasting

**DOI:** 10.3389/fneur.2022.779551

**Published:** 2022-02-11

**Authors:** Aria Moss, Evan Moss, Robert Moss, Lisa Moss, Sharon Chiang, Peter Crino

**Affiliations:** ^1^Northern Virginia Community College, Alexandria, VA, United States; ^2^W. T. Woodson High School, Fairfax, VA, United States; ^3^Seizure Tracker, LLC, Springfield, VA, United States; ^4^TSC Alliance, Silver Spring, MD, United States; ^5^Department of Neurology and Weill Institute for Neurosciences, University of California, San Francisco, San Francisco, CA, United States; ^6^Department of Neurology, University of Maryland School of Medicine, Baltimore, MD, United States

**Keywords:** epilepsy, seizures, forecasting, devices, patient perspective, tuberous sclerosis complex, seizure detection

## Introduction

My name is Aria Moss. My brother, Evan Moss, was diagnosed with epilepsy when he was 3 months old, and Tuberous Sclerosis Complex (TSC) at 2 years. TSC is a genetic disorder that causes tumor growth in vital organs; Evan has tumors in his brain, kidneys, liver, and skin. At 4 years old, he was having >15 seizures a day and underwent brain surgery, becoming seizure-free for 2 years. Unfortunately, his seizures recurred at age six, as nocturnal status epilepticus. He's since had multiple surgeries and tried >10 medications to treat them. While Evan's seizures are now less frequent, occurring every 2 weeks rather than daily, their unpredictability and impact have remained.

Evan is currently 17 years old. I'm nineteen with hopes of 1 day becoming a doctor. Our parents are both highly involved with the epilepsy community: our mom, Lisa, works for the TSC Alliance, and our dad, Rob, developed the online seizure diary Seizure Tracker.

The following interview, conducted by Evan's neurologist, Dr. Peter Crino, shares the perspective of our family.

## Detection Device Functionality

The features of detection devices valued by patients as end-users are vital to device design. While important features may vary from user to user, certain features are considered particularly important by the majority of patients. High comfort, low visibility, and high usability are often crucial features for patient uptake (1), whereas uncomfortable or visible devices dramatically limit patient willingness to use them and are weighed against benefit and reliability (2).

Dr. Peter Crino: “Today we're meeting with Evan Moss, along with his parents, Rob and Lisa, and sister, Aria. We'll touch on how Evan and his family deal with the uncertainty of living with seizures and how seizure detection/forecasting carries for their family hope for ultimately improving quality of life. There are many emerging approaches for seizure detection and forecasting that promise individuals with epilepsy a view of when seizures might occur (3, 4). All are exciting but imperfect (5). Evan, what are your experiences with seizure detection devices? What's worked for you, what have you struggled with?”

Evan Moss: “Well, a lot of the devices haven't helped me much. My seizures cycle every two weeks. I can tell on my own when I'm getting close to a seizure, but I don't know exactly when it'll occur. I need to know ahead, and I need precision; I want to know what day it's going to be on.”

Dr. Crino: “That's an important observation. As you said, seizure detection and forecasting are distinct. Many individuals with epilepsy have some ability to self-predict, but it's not always reliable, and doesn't work for everyone (6, 7). Alerting devices say when you have a seizure but knowing exactly when seizures will occur would really help you to prepare. You've tried several devices for seizure alerting–a pulse oximeter, heart rate patches, and cameras, for example. What have been your experiences with those?”

Robert Moss: “When we used the pulse oximeter and heart rate patch, they weren't super reliable. Other devices, like EMG, relied on algorithms that required lengthy periods of muscle involvement. Evan's seizures included muscle contractions that were detected by EMG but wouldn't last long enough for it to alert.”

Lisa Moss: “There were also challenges wearing the pulse oximeter; when he wore it on his hand or foot, it didn't stay on. The heart rate patches stuck to his chest and were difficult to remove. They'd irritate his skin.”

Dr. Crino: “So they were uncomfortable, which led to a lack of functionality.”

Rob: “Comfort and reliability have definitely been an issue. The best for us as parents has been video monitoring. We can tell based on motion whether Evan's having a seizure, and run in to treat it.”

Evan: “I think there are four features needed for a good device: it needs to be comfortable, easy to use, accurate, and precise. They're different–precision is always hitting the same spot; accuracy is always hitting the bullseye.”

## Privacy and Stigma

When using wearable devices, patients often report that a device's visibility and privacy (or lack thereof) negatively impacts their experience, which can be isolating or uncomfortable (1, 2, 8). Here, the Moss family reflects on similar experiences with other seizure detection methods they use.

Dr. Crino: “Evan, what are your thoughts about being monitored on camera for seizures? Is that comfortable for you?”

Evan: “Not at all! There's a camera constantly on in my room. I have a hard time ever feeling that I'm not being watched.”

Dr. Crino: “I think most people would be pretty opposed to having a camera in their room, right? We hear this in epilepsy monitoring units; you're live theater, all the time. It serves a function— you all can have eyes on Evan and respond rapidly, which is great, but privacy is an big issue (9). Tangentially—tell me about your experience with Mindy, your seizure alert dog. Is it extra attention you don't want?”

Evan: “It's not that bad for me. At school, everyone knows I have Mindy and love her! I can also choose to leave her at home on small outings.”

Lisa: “There are even challenges with having Mindy. You may think everybody knows service dogs are allowed in public spaces but that isn't always the case. We've had to fight to enter restaurants, doctor's offices, even hospitals!”

Dr. Crino: “Really?”

Lisa: “Yes! Life can become confrontational doing basic things, and it can be unsettling. We're a spectacle–everywhere we go, heads turn, people comment. As soon as the vest is on, you get that attention, whether you want it or not.”

Rob: “Our community's looking for solutions, but I feel like there's rarely conversation about the unintended consequences. Sometimes the lifestyle changes have an even bigger detrimental effect on quality of life.”

Lisa: “I think we were really hoping for independence for Evan, so he could sleep alone without the camera. Mindy alerts two days ahead of a seizure and responds as it happens, but we usually wake up before she does. She's part of our team but doesn't fill all the gaps.”

## Forecasting Horizons and Priorities

Many surveys on patient needs with regards to the performance of seizure forecasting algorithms have been conducted. In general, patients have reported that high sensitivity is vital for seizure prediction (10), with the potential morbidity/mortality from false negatives a high concern (11). One survey of 1,168 people with epilepsy found that “detecting all seizures” was rated as the most important priority for detection devices (12). Another survey found that about 60% of people and caregivers of epilepsy required 100% accuracy in detection rates to find devices useful (13). Only 25% (32%) of patients have said they would be very likely to continue using a forecasting tool if it made one false negative (false positive) prediction, respectively (14).

Dr. Crino: “Let's discuss seizure prediction. Recent evidence suggests that when you have a seizure, it's been building up for hours, maybe days (15). There're lots of markers that go up in the blood and brain around the time of seizure activity (16, 17). It's clearly a systemic effect, we just don't have a way to reliably pick it up in advance.”

Lisa: “It would be great if there was a way to predict a seizure and treat preventatively so it doesn't occur or you're prepared. If you're going to go out and you're alerted about a likely seizure, maybe plans change. Evan's needs are different since his seizures happen in his sleep.”

Rob: “That's why patients have to be involved in developing devices. Electrographic seizures are considered a gold standard in prediction, but as a father I don't want to predict every seizure. I want to know when he'll have a seizure that would require intervention.”

Dr. Crino: “There's discussion by developers of what prediction horizons they should aim for. For you, what would be a good prediction horizon?”

Evan: “My seizures are in my sleep, and usually happen early in the morning. If I get an alert too far ahead, like over two hours, I'm going to lie awake, dreading a seizure. But it needs to be far enough ahead to act, at least more than 10 min.”

Dr. Crino: “Earlier, Evan gave four important features of a seizure forecasting device–ease of use, comfort, precision, and accuracy. Is there anything else?”

Rob: “It's dependent on the characteristics of each person's epilepsy. There was a switch when Evan went from many small seizures a day to status seizures every two weeks. Until we had to act on those long seizures, we didn't care about predicting the small ones. We already knew he was going to have ten or fifteen seizures the next day.”

Aria Moss: “In general, a device has to be able to predict seizures better than the patient or caretaker can–which isn't easy. But until you can replace their usual detection methods, you're not going to alleviate meaningful burden on the family. Evan has seizures every two weeks, which always go into status and are treated with emergency medication right away, and we often call 911. We can't miss a seizure. If we have a device that works 95% of the time but will still miss 5% of his seizures, that's just not helpful–it's not enough to rely on alone.”

Dr. Crino: “That's an interesting point–your family has put together a strategy for 100% catch rate. That's the benchmark for these devices: 100% sensitivity. You really can't miss any seizures; there's morbidity associated with that.”

## Discussion: The Patient Perspective

About a fifth of patients have expressed that existing devices are not effective for their seizure type(s), which causes skepticism about the use of forecasting or detection devices (14). Almost all evidence for devices currently on the market is specific to seizure types with major motor features, and there is little evidence on accuracy for other seizure types (1). Ultimately, in order to develop effective devices, it is vital to ensure patient voices are considered throughout the development process.

Dr. Crino: “I'd like to wrap up with a broader question. What's your perspective on how well the research community is involving patient perspectives?”

Lisa: “I don't think that the patient voice is brought in early enough. The patient is the one to buy the device, follow the method, wear the tool. When patients are involved early on, they share their views, and some aspects might improve. Sometimes a change will evade developers because they're not living it day to day, so it's important to have a diverse population of patients involved in those discussions. This is related to patient-doctor interactions, too; patients need to be viewed as valuable and knowledgeable members of the treatment team and be included in all aspects of care. As parents we've seen our son have thousands of seizures, and that provides expertise. We're experts in Evan's epilepsy and which treatments will improve his quality of life. It's the same when considering a seizure detection device: it's important to recognize the knowledge of the patient community in all stages of development.”

Rob: “Epilepsy is a hard disorder to manage and requires strong patient/physician communication to treat effectively. Technology needs to compliment that communication, not replace it. Developing technologies that collect data alongside patient-reported outcomes is a way developers can prioritize both clean data collection and patient interests.”

Dr. Crino: “I think you're right–many people think about this problem in terms of removing the patient altogether, but to be honest with you, I don't foresee a future where we can do that. The patient tells us what outcomes matter most. Someday, maybe seizure forecasting will be tied to adjusting the wiring in the brain in real-time (18). We don't have any therapies right now to adjust the wiring in the brain, to really help patients. That's for you to figure out when you're a doctor, Aria.”

Aria: “Talking about predicting seizures is amazing; it's a new frontier for epilepsy. When I'm a doctor, I hope we have solutions that go beyond anything we have today. I'm excited to see where we'll be ten, twenty years down the line.”

## Author Contributions

Manuscript conceptualization was developed by SC, AM, and RM. The interview was conducted with PC as interviewer and AM, EM, LM, and RM as interviewees. AM drafted the manuscript. All authors read, refined, and approved the manuscript.

## Conflict of Interest

RM is the cofounder/owner of Seizure Tracker, LLC and reports financial income to Seizure Tracker, LLC, from Neurelis, UCB, Greenwich Biosciences, Neuropace and the Tuberous Sclerosis Complex Alliance. SC is supported by the National Institute of Neurological Disorders and Stroke, National Institutes of Health (5R25NS070680-12). The remaining authors declare that the research was conducted in the absence of any commercial or financial relationships that could be construed as a potential conflict of interest.

## Publisher's Note

All claims expressed in this article are solely those of the authors and do not necessarily represent those of their affiliated organizations, or those of the publisher, the editors and the reviewers. Any product that may be evaluated in this article, or claim that may be made by its manufacturer, is not guaranteed or endorsed by the publisher.
